# Teeth syndrome: diagnosis, complications and management

**DOI:** 10.11604/pamj.2015.22.71.7313

**Published:** 2015-09-28

**Authors:** Mohamed Ali Sbai, Sofien Benzarti, Monia Boussen, Riadh Maalla

**Affiliations:** 1 Orthopedic Surgery and Trauma Department, Maamouri Hospital, Nabeul, Tunisia; 2 Emergency Department, Mongi Slim Hospital, Tunis, Tunisia; 3 Plastic surgery Department, La Rabta Hospital, Tunis, Tunisia

**Keywords:** Human bite, cellulitis, arthritis, finger, Hand surgery

## Abstract

Teeth syndrome or fight bite is a specific entity in hand surgery that is little known. It includes infectious complications of the hand following a fist against the teeth. Neglected or misdiagnosed this injury frequently leads to serious complications that could compromise the function of the hand. A retrospective study was performed on 20 patients treated for teeth syndrome at our department, during a period of 12 years (January 2003 to April 2015). All young adults with a mean age of 28 years and a significant male predominance. The dominant side was involved in 15 patients. Lesions were divided into 4 cases of simple dorsal wounds facing the MP joint, 8 cases of dorsal hand cellulitis, and 8 cases of arthritis and osteoarthritis of the metacarpophalangeal (MP) joint of the long fingers. The index was the most affected finger. Treatment consisted in debridement of necrotic tissues, stabilization with external fixation for arthritis, skin reconstruction was performed secondarily. Result was assessed as good in 60% of cases. Clenched fist injuries to the mouth (teeth syndrome or fight bite) are known as being the worst human bites. Usually treated as minor injuries, without realizing a breach of the joint capsule, a lesion of the extensor tendon, or a contamination by oral flora. Any patient with a wound near the joint of the hand and was involved in a fight, need an appropriate evaluation and a specialized treatment to avoid serious complications.

## Introduction

**Figure 1:**
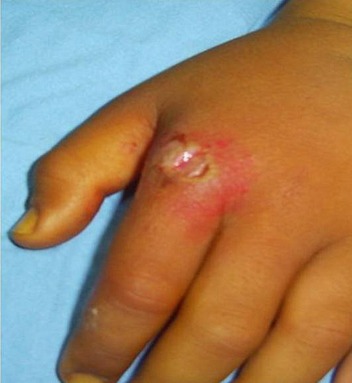
Infected wound on the dorsum of the left hand in front of the MP joint of the index following a fist against the teeth

The teeth syndrome or fight bite is a specific entity in hand surgery that is little known. It includes infectious complications of the hand following a fist against the teeth and gathers the dorsal hand cellulitis, arthritis and osteoarthritis of the metacarpo-phalangeal (MP) joints of the long fingers of the hand (Figure 1). Neglected or misdiagnosed this injury frequently leads to serious complications that could compromise the function of the hand [1]. We present a serie of 20 patients with teeth syndrome. The objective of this work is to recall this little known syndrome and insist on early and specialized care to avoid serious complications.

## Methods

We performed a retrospective study of 20 patients treated in our department, during a period of 12 years (January 2003 to April 2015). Epidemiological data, clinical features, investigations, treatment and follow up of the patients with this type of injury were analyzed.

## Results

**Figure 2:**
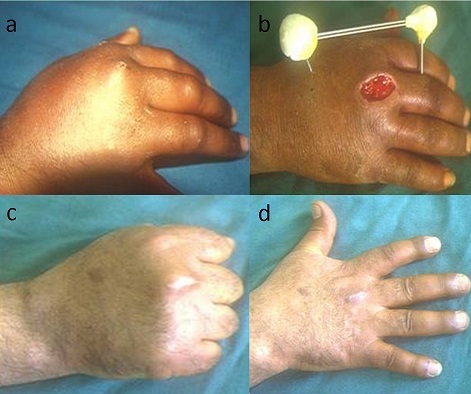
Arthritis stage 2 of the MP joint of the right middle finger (a), treated by excision and stabilization by Beaubourg's external fixator (b), provided healing with a good functional and aesthetic result (c, d)

**Figure 3:**
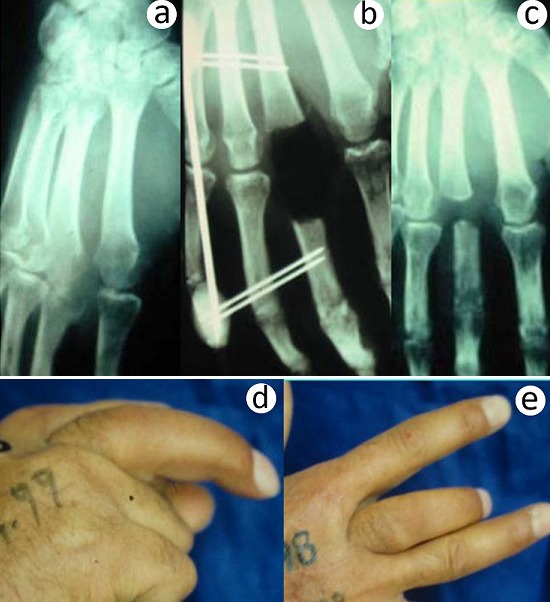
Osteoarthritis of the MP joint of the right middle finger (a), treated by excision, arthrectomy and stabilization by Beaubourg's external fixator (b), provided healing with a bad functional and aesthetic result (c, d, e)

**Figure 4:**
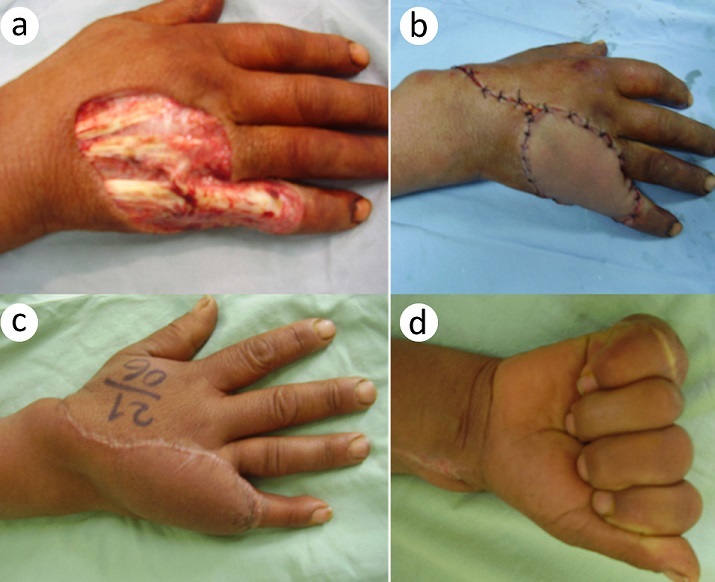
Dorsal cellulitis of the right hand complicating an infected wound of a fist against the teeth, next to the MP joint of the little finger in a diabetic patient. She was treated by excision (a), and a secondary radial forearm flap coverage (b), provided good functional and aesthetic result (c, d)

Most of our patients are young adults; the mean age is 28 years with extremes of 16 and 42 years. A clear male predominance was noted with 18 males. Two of our patients are diabetics, the dominant side is involved in 15 cases. Lesions were divided into 4 simple dorsal wounds facing the MP joint, 5 cases of arthritis of the MP joint (Figure 2), 3 cases of osteoarthritis of the MP joint (Figure 3) and 8 dorsal hand cellulitis (Figure 4). The index is the most involved finger (Table 1). A plain radiograph of the hand is systematically requested for the research of bone injury. The patient presentation period was 24 hours to 15 days with an average of 60 hours. All patients were operated under general anesthesia. They received a surgical excision, and a stabilization of the MP joint by an external fixator for arthritis (Figure 2, Figure 3). A bacteriological study was performed in all patients showing a predominance of gram negative bacilli bacteria. Antibiotic chemotherapy was immediately started for all patients then adapted according to the antibiogram. Three patients presented a section of the extensor system that was secondarily repaired in two cases. Loss of skin substances received flap coverage in 8 cases, 3 patients required delayed closure and spontaneous healing for 9 patients. All patients underwent a physiotherapy program. The delay of returning to work was 60 days. The results were evaluated using a score that considers digital mobility and aesthetics of the hand; we obtained 60% of good results (Table 2).

**Table 1:** Distribution of the fingers by the type of injury

	wound	cellulitis	Arthritis stage 1	Arthritis stage 2	osteoarthritis	Total
index	1	3	1	1	1	7
Middle finger	1	1	0	1	1	4
Ring finger	1	2	0	0	1	4
Little finger	1	2	1	1	0	5
Total	4	8	2	3	3	20

**Table 2:** Distribution of the functional results by the type injury

	Good result	Average result	Bad result	total
wound	4	0	0	4
cellulitis	5	3	0	8
arthritis	3	2	3	8
total	12	5	3	20

## Discussion

Human bites represent about 2% of all bite injuries to the hand [2]. Clenched fist injuries to the mouth (teeth syndrome or fight bite) are a frequent cause, known as being the worst human bites. They are usually treated as minor injuries, without realizing a breach of the joint capsule, a lesion of the extensor tendon, or a contamination by oral flora. Any patient with a wound near the joint of the hand and was involved in a fight, need an appropriate evaluation. A tooth mark at the inoculation site is often noted [3]. Teeth syndrome has to be properly treated because of it serious complications and significant morbidity [4]. The human mouth contains a mixed flora of about 50 species of aerobic and anaerobic bacteria, with the most frequent ones being respectively Staphylococcus aureus, Streptococcus, Corynebacterium, and Eikenella corrodens [5]. The incrimination of Eikenella corrodens has been correlated in many series to a delayed presentation [6–8]. Callaham, found in his study a high rate of complications (septic arthritis and osteoarthritis) associated with a delayed presentation of teeth syndrome [2, 9]. Based on the epidemiological features, broad-spectrum antibiotics are usually started with combined therapies, while waiting for bacteriological results [4]. The Management of teeth syndrome injuries consist in an exploration of the wound in the operating room with thorough irrigation of the joint if joint capsule found to be breached and debridement of the bite injury. Hospitalisation for 48 hours and administration of intravenous antibiotics is necessary [10, 11].

Septic arthritis and osteoarthritis are very serious complications requiring a specialized urgent care. We systematically practice excision of the inoculation site, joint irrigation for arthritis stage 1, synovectomy and stabilization with external fixation for stage 2, arthrectomy and stabilization by external fixation for osteoarthritis. For stabilization in the arthritis, We prefer the Beaubourg's external fixation because of its simplicity, availability and cost [12].

Cellulitis of the hand initially requires excision and secondarily a limited tissue loss could be left for spontaneous healing, major skin defects exposing the joints or tendons are covered by flaps.

Wounds associated with injuries of the extensor tendon should be thoroughly irrigated and to avoid a delayed rupture, any section of more than 50% should be immediately repaired [13, 14].

## Conclusion

The teeth syndrome remains little known and neglected, progression to septic arthritis of MP joints is serious and causes impairment and loss of hand functions. Appropriate early treatment of teeth syndrome is the key to success. Treatment with antibiotics, surgical drainage, debridement and copious irrigation, proved to be effective. An early and appropriate care is essential to avoid serious complications.
